# Potential of Entomopathogenic Nematodes to Control the Cabbage Stem Flea Beetle *Psylliodes chrysocephala*

**DOI:** 10.3390/insects14070665

**Published:** 2023-07-24

**Authors:** Claire Price, Heather Campbell, Tom Pope

**Affiliations:** Agriculture and Environment Department, Harper Adams University, Newport, Shropshire TF10 8NB, UK; hcampbell@harper-adams.ac.uk (H.C.); tpope@harper-adams.ac.uk (T.P.)

**Keywords:** oilseed rape pests, CFSB, entomopathogens, biopesticides

## Abstract

**Simple Summary:**

Cabbage stem flea beetles were controlled through the use of neonicotinoid insecticides to protect oilseed rape crops grown in the UK and other parts of Europe. However, since the ban of this class of insecticide for this use in 2013, the only authorized insecticides to control the cabbage stem flea beetle are pyrethroids, which are toxic to other insects and to which cabbage stem flea beetles have developed resistance. New control solutions are necessary, such as the use of entomopathogenic nematodes. Here we evaluated the potential of several entomopathogenic nematode species to control the adult cabbage stem flea beetle in the laboratory. Results are encouraging, with high flea beetle mortality obtained, i.e., 75% with *Heterorhabditis bacteriophora*, 80% with *Steinernema feltiae*, 85% with *Steinernema carpocapsae* and 70% with *Steinernema kraussei* as opposed to the control treatment which led to 23% mortality. Next, we tested whether entomopathogenic nematodes were compatible with different adjuvants that may protect nematodes against detrimental UV radiation and desiccation. All adjuvants tested were found to be compatible with nematodes under the laboratory conditions used. This study is a first step in developing an effective and safe solution to control the cabbage stem flea beetle in oilseed rape crops.

**Abstract:**

Cabbage stem flea beetle (CSFB) is an important pest of oilseed rape that was controlled by neonicotinoid seed treatments until they were banned for this use in 2013. Since then, CSFB has been a difficult pest to control, partly due to widespread resistance to pyrethroid insecticides. Alternate solutions are necessary. Here, four entomopathogenic nematode (EPN) species were tested against CSFB adults under laboratory conditions. In addition, a bioassay was completed to test for EPN compatibility with a range of adjuvants (glycerin, xanthan gum and flame retardant) to protect EPNs from UV radiation and desiccation. Results show that EPNs have the potential to control CSFB adults under laboratory conditions. *Heterorhabditis bacteriophora* caused 75% CSFB mortality at a concentration of 4000 nematodes/mL after six days, *Steinernema feltiae* caused 80% CSFB mortality when applied at a concentration of 40,000 nematodes/mL after two days, *Steinernema carpocapsae* caused 85% mortality at a concentration of 10,000 nematodes/mL after six days, and *Steinernema kraussei* caused no more than 70% CSFB mortality overall compared to the water control, which led to 23% mortality. *Steinernema feltiae* and *H. bacteriophora* survival was 100% when exposed to adjuvants, except *S. feltiae* with glycerin and *H. bacteriophora* with flame retardant. Further research to evaluate the efficacy of EPN and adjuvants under field conditions is necessary.

## 1. Introduction

Oilseed rape (*Brassica napus*, Linnaeus) is the third most widely grown and the fourth most productive crop (in terms of tonnes/ha) grown in the United Kingdom (UK) [1]. The production and value of oilseed rape in the UK has, however, decreased over recent years due largely to the increasing threat of pests, such as cabbage stem flea beetles (CSFB, *Psylliodes chrysocephala* Linnaeus) (Coleoptera: Chrysomelidae) [2]. This has led to the area of oilseed rape grown to decrease from 756,000 hectares in 2012 to 307,000 hectares in 2021 [1].

Cabbage stem flea beetles invade oilseed rape at crop establishments, where they are often the most important pest species [3,4,5,6,7]. Damage by adults in the autumn can lead to seedling death [8], and damage by larvae can lead to stem wilting, delayed flowering, higher susceptibility to frost damage [4] and pathogens [9,10,11], or total plant collapse [4,12,13,14,15,16,17]. 

Until their ban by the European Union in 2013 [18], neonicotinoid insecticides applied to oilseed rape crops as a seed dressing were the primary means of protecting plants against CSFB [19]. Since 2013, pyrethroid insecticides applied as a foliar spray have been the only conventional synthetic insecticide option that oilseed rape growers have had for the control of CSFB. The overreliance on pyrethroid insecticides threatens non-target organisms such as pollinators and natural enemies [17], and has led to development of widespread resistance to this type of pesticide in CSFB populations [20,21].

One alternative to the current reliance on synthetic pesticides is the use of bioprotectants [22]. The term bioprotectant includes crop protection tools such as semiochemicals (substances emitted by plants, animals, and other organisms), microbials including bacteria, fungi, protozoans, viruses, and invertebrate biocontrol agents/macrobials, which include entomopathogenic nematodes (EPNs). Along with entomopathogenic fungi, EPNs have been described as the organisms with the greatest potential to provide effective control of oilseed rape pests [23]. EPNs, in particular, are attractive options with which to control a pest such as CSFB, as they actively search for their host once applied to the crop, present no harm to vertebrates and can kill an individual insect even when nematodes are applied at very low densities [24].

EPNs in the nematode genera *Steinernema* and *Heterorhabditis* are used to control many pest insects, such as the soil-dwelling larvae of leafminers, thrips, craneflies, garden chafers and various species of moth and weevils [25]. There are three juvenile stages of EPNs, but the only free-living stage is the third-stage juvenile, also known as the infective juvenile (IJ), that searches for and infects a host. The IJ enters the host through natural openings (mouth, anus and spiracles), or by piercing the cuticle in the case of *Heterorhabditis* spp. with the help of an anterior tooth [26,27] and reaches the haemolymph [28]. Once within the haemolymph, the IJ releases bacteria that live in the gut of the nematodes and with which they have a mutualistic, symbiotic relationship: *Photorhabdus* sp. for species of *Heterorhabditis* and *Xenorhabdus* sp. for species of *Steinernema* [29,30]. Once released into the haemolymph, the bacteria proliferate and kill the insect through septicemia and physical action within 24–72 h. The digested tissues provide a food source for the IJs, which develop into adult nematodes and reproduce. Depending on the size of the host, two or more generations of nematodes can develop in the same cadaver [25], but once nutrients are exhausted, the next generation of IJs will begin to search for a new host [31]. 

Despite being highlighted as having the greatest potential to provide the effective control of oilseed rape pests [23], in a recent laboratory study, it was concluded that EPNs are not effective against CSFB adults [32]. This conclusion was reached after testing the efficacy of Nemaplus^®^ (*Steinernema Feltiae* (Filipjev)), Nematop^®^ (*Heterorhabditis bacteriophora* (Poinar)) and Nemastar^®^ (*S. carpocapsae* (Weiser)) against CSFB adults. Each EPN product was tested at a concentration of 2000 nematodes/mL by pipetting 1ml of the nematode suspensions into Petri dishes filled with sand (7% RH) before adding CSFB adults. Despite the current uncertainty around their effectiveness, testing EPNs under laboratory conditions is necessary as a first step in determining if the pest is susceptible to these control agents. Importantly, this can be performed where confounding variables such as biotic and/or abiotic factors can be excluded [32]. A clearer picture on the efficacy of EPNs against flea beetle pests of brassica crops is required as, to date, most studies have been performed under field conditions. This includes work on the striped flea beetle (*Phyllotreta striolata* (Fabricius)) [33,34,35,36,37,38], the crucifer flea beetle (*Phyllotreta cruciferae* (Goeze)) [39,40] and an unnamed *Phyllotreta* species [23,41,42]. The results from these studies have been variable and have not been based on results from preliminary laboratory work to evaluate the potential of the EPN species tested.

EPNs are sensitive to UV radiation and desiccation [43,44] and so their effective use under field conditions requires that these organisms are protected from these abiotic factors. The use of adjuvants in combination with EPNs against flea beetles has previously been tested in canola fields [45,46] and is one way in which limitations on the use of EPNs can be overcome. Glycerin has been reported to be an antidesiccant and may help nematodes to persist on foliage for longer [47]. Xanthan gum has been reported to prevent nematode sedimentation and as such may improve application to crops [48]. Finally, flame retardants have been reported to protect nematodes against abiotic factors and enhance their efficacy against flea beetles and other pests [45,46,49,50]. 

Here, we investigate the efficacy of commercial formulations of the EPNs *Steinernema feltiae* (Nemasys), *Steinernema carpocapsae* (Nemasys C), *Steinernema kraussei* (Steiner) (Nemasys L) and *Heterorhabditis bacteriophora* (Nemasys H) against CSFB adults under laboratory conditions. These species of nematode were chosen for their ability to remain active at low temperatures [51,52], a useful quality in oilseed rape crops grown in the UK, as the pest establishes in the autumn or spring [53]. The objective of this study was to determine whether the selected EPN species are suitable candidates for further work under semi-field and field conditions.

## 2. Material and Methods

### 2.1. Insects and Plants

CSFB adults were collected in July 2019, 2020, and 2022, at harvest from farms in Shropshire, UK. The insects were kept in ventilated mesh cages (30 × 30 × 30 cm) in a controlled environment room (Fitotron^®^ SGR 122, Weiss Technik UK Limited, Loughborough, UK) at a constant 20 °C temperature, 60% RH and 16/8 h day/night cycle and fed by placing potted oilseed rape plants (variety Mirakel) grown under glasshouse conditions to growth stage 12 (BBCH system) into each cage. Potted oilseed rape plants were replaced every two weeks. Insect populations were kept under these conditions for up to three months before being used in a bioassay. The sex of the tested individuals was not determined before the bioassays and beetles were taken straight from the cages for bioassays. Fully expanded first and second true leaves were used as a food source for CSFB in the bioassays. The leaves were collected from young potted oilseed rape plants (variety Mirakel) grown under glasshouse conditions and that had reached a minimum growth stage of 12 (BBCH system). A water treatment was included alongside nematode treatments in each bioassay as a control. Tap water pH was 7 and hardness (CaCO_3_) was 425 parts per million.

### 2.2. Entomopathogenic Nematodes

Commercial formulations of EPN (see Table 1) were supplied by BASF Agricultural Solutions UK (Littlehampton, UK). Packs of EPN were kept refrigerated at 5 °C until use and were used within the stated use-by date.

### 2.3. Mortality Bioassay

Here, the four EPN species were each tested at three concentrations of live nematodes: 4000, 10,000, or 40,000 nematodes/mL. Each concentration was replicated three times. EPN suspensions were prepared by suspending a commercial formulation of nematodes in 1.5 L of tap water (stock suspension). EPNs were activated by vigorously stirring the stock suspension for five minutes, then a 1 mL sample was taken from the stock suspension using a micropipette. This 1 mL aliquot was then diluted by adding it to a conical flask containing 200 mL of tap water. From this dilution, 1 mL was pipetted into each of the three 90 mm diameter Petri dishes and additional water was added to each Petri dish to create a thin film of water over the base of the dishes. The number of active nematodes in each Petri dish was counted using a stereo microscope at magnification × 40 (Microtec Microscopes LTD, Somerset, UK). Using the mean number of live nematodes from the three Petri dishes, the number of live EPNs in the stock suspension was calculated and dilutions required to create the desired concentrations for the bioassays completed. 

The following method was adapted from published literature [54,55]. Petri dishes (6 cm diameter) were prepared with two layers of filter paper (Whatman No. 1, Cytiva, Marlborough, MA, USA). One millilitre of each nematode suspension (or water for the control) was applied to the filter paper in each Petri dish with a micropipette. Then, ten CSFB adults were added per dish. A small piece of oilseed rape leaf was included in each dish as a source of food. Once prepared, the Petri dishes were placed in a fully randomised design inside a controlled environment cabinet set to a constant 25 °C, 60% RH and 16/8 h day/night cycle (SL2/RH, LEEC Ltd., Nottingham, UK). 

CSFB mortality was assessed every two days for a period of eight days by counting the number of dead beetles in each dish. As CSFB feign death when threatened, death was confirmed when the individuals were not moving when ‘prodded’ repeatedly with a dissecting needle for more than ten seconds, and when the beetles’ legs were in a splayed position. Moribund insects were considered dead. 

### 2.4. Compatibility between Entomopathogenic Nematodes and Adjuvants

Nemasys (*S. feltiae*) and Nemasys H (*H. bacteriophora*) were used in this bioassay and each species was tested in combination with each adjuvant. The concentrations of the used adjuvants are listed in Table 2. Each adjuvant was dissolved in tap water.

Commercial formulations of each EPN species were diluted separately in 5 L of tap water. IJs were activated by oxygenating using an aquarium pump for ten minutes prior the start of the bioassay and all the way until the end of the bioassay, and the 5 L bottle was regularly shaken to prevent nematode sedimentation and keep the suspension homogenised. Petri dishes (8.5 cm diameter) were lined with filter paper (Whatman No. 1, Cytiva, Malborough, MA, USA). In each Petri dish, approximately 5000 IJs were mixed with one of the adjuvants in 1ml of water (only nematodes for the water control) and were added with a micropipette after thorough agitation of the suspension. The Petri dishes were then sealed with paraffin film (Bemis^TM^ Parafilm^®^, Neenah, WI, USA) to prevent EPN from escaping, and wrapped in tin foil to protect nematodes from UV radiation emitted by the light bulbs. The dishes were then placed in incubation chambers (round plastic boxes, 12 cm/7 cm diameter top/bottom, 6 cm height, lined with four layers of wet paper towel) to maintain high humidity. All incubation chambers were placed in a growth room (Fitotron^®^ SGR 122, Weiss Technik UK Ltd., Loughborough, UK) with a 16/8 h day/night photoperiod and 20 °C for seven days. Two data loggers (Tempo Disc, Blue Maestro, London, UK) were placed in similar conditions two days prior to the bioassay to confirm that these conditions resulted in 100% humidity. Concentrations for xanthan gum were lower (see Table 2) than for other adjuvants due to the highest concentration of 10% not completely dissolving in water.

After seven days, the Petri dishes were opened, and each disc was rinsed into a second Petri dish using a squeeze bottle filled with tap water. Then, the first 30 nematodes counted within a consecutive series of fields of view were scored as either being dead or alive based on their response when repeatedly probed with a dissecting needle.

### 2.5. Statistical Analysis

Data were analysed in R (version 4.2.2) and RStudio (version 2022.12.0). CSFB mortality was analysed using mixed effect models from the package lme4 [56]. EPN survival when exposed to adjuvant was analysed using one-way ANOVA on a linear model of the data. Significance groups were computed using the cld(lsmeans()) function included in the packages multcomp [57] and lsmeans [58], or using the HSD.test() function included in the package agricolae [59]. Graphical illustrations for CSFB mortality were made using the geom_col function from the package ggplot2 [60] after the data were tidied with the mutate function from the package tidyverse [61]. Graphical illustrations for EPN survival with adjuvants were made with the boxplot function from the package graphics [62] after the data were tidied with the mutate function from the package tidyverse. 

## 3. Results

### 3.1. Mortality Bioassay

**Figure 1:**
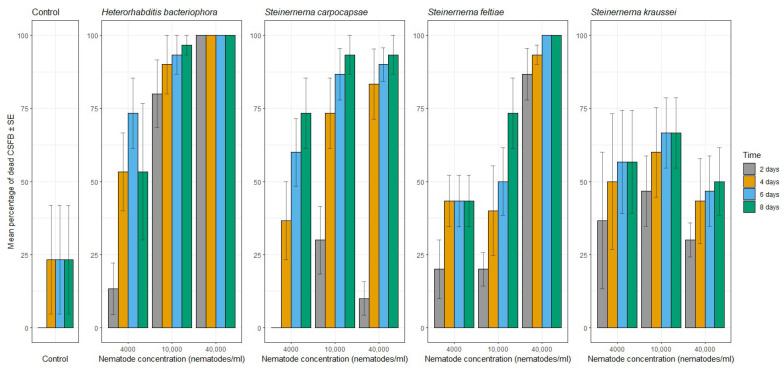
Mean percentage of dead cabbage stem flea beetle (CSFB) ± SE after two, four, six and eight days of contact with four different species of entomopathogenic nematodes (EPNs) (concentrations of nematodes/mL) or water (control). Values are cumulative over the days.

CSFB mortality results are illustrated in Figure 1. All EPN treatments resulted in significantly higher mortality than was caused by the water control (F = 7.14, df = 4, *p* < 0.001). There was no difference in CSFB mortality between the two highest concentrations (t = −1.18, df = 32, *p* = 0.247) and both caused significantly higher mortality than the lowest concentration and the control (t = −3.78, df = 32, *p* < 0.001). Overall, CSFB mortality increased with time (F = 38.56, df = 3, *p* < 0.001).

### 3.2. Compatibility between Entomopathogenic Nematodes and Adjuvants

*Steinernema feltiae* survival was significantly lower when EPNs were exposed to glycerin compared with other adjuvants and the control (F = 6.308, df = 3, *p* = 0.001) whereas *H. bacteriophora* survival was significantly lower when EPN were exposed to the flame retardant compared with other adjuvants and the control (F = 9.684, df = 3, *p* < 0.001).

**Figure 2:**
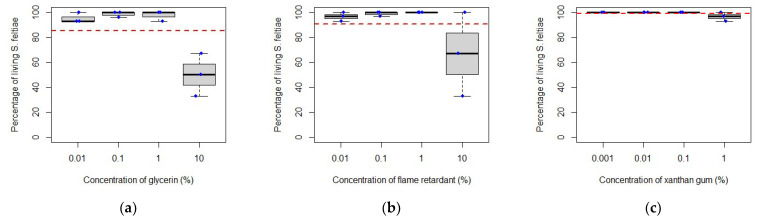
Percentage of living *Steinernema feltiae* after exposure to various concentrations of glycerin (**a**), flame retardant (**b**) and xanthan gum (**c**). The red line represents the overall mean of the data. The blue dots help visualize various data points.

**Figure 3:**
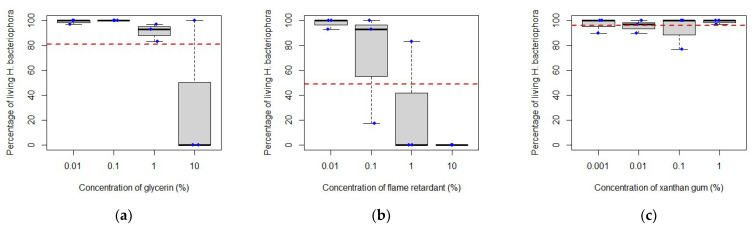
Percentage of living *Heterorhabditis bacteriophora* after exposure to various concentrations of glycerin (**a**), flame retardant (**b**) and xanthan gum (**c**). The red line represents the overall mean of the data. The blue dots help visualize various data points.

Following these observations, the results for each adjuvant were analysed separately for each EPN species (Figure 2): there was no significant difference in *S. feltiae* survival when exposed to 0.01, 0.1, 1 or 10% of flame retardant (F = 2.702, df = 3, *p* = 0.116) and to 0.001, 0.01, 0.1 or 1% of xanthan gum (F = 2.703, df = 3, *p* = 0.116); however, when exposed to 0.01, 0.1, 1 or 10% of glycerin (Figure 2), survival was significantly lower at the highest concentration (F = 20.53, df = 3, *p* < 0.001). There was no significant difference in *H. bacteriophora* survival when exposed to the same concentrations of glycerin (F = 3.609, df = 3, *p* = 0.065) and to the same concentrations of xanthan gum (F = 0.384, df = 3, *p* = 0.767) but when exposed to a range of concentrations of flame retardant (Figure 3), survival decreased progressively with an increasing concentration of this adjuvant; EPN survival was significantly different between each concentration except between 0.1% and 1% (F = 5.11, df = 3, *p*-value = 0.029). 

## 4. Discussion

### 4.1. Mortality Bioassay

After two days, *Steinernema feltiae* caused 80% CSFB mortality when applied at the concentration of 40,000 nematodes/mL compared to 23% mortality in the water control treatment. *Heterorhabditis bacteriophora* caused approximately 75% CSFB mortality recorded after six days when a concentration of 4000 nematodes/mL was applied. *Steinernema carpocapsae* was effective at a concentration of 10,000 nematodes/mL when 85% mortality was recorded six days post nematode application. *Steinernema kraussei* was the least effective species tested here, with no more than 70% CSFB mortality recorded for any of the concentrations tested.

A previous laboratory study has investigated the efficacy of EPN against CSFB adults. In this earlier work [63], 25% adult CSFB mortality was recorded with *S. carpocapsae*, 16% CSFB mortality with *S. feltiae* and 7% CSFB mortality with *H. bacteriophora*, and only *S. carpocapsae* was found in dead CSFB. The authors concluded that CSFB adults were not the most appropriate growth stage to be targeted using EPN and indeed results from this study against CSFB larvae were more encouraging. Differences in adult CSFB mortality between the results recorded in previous work and the results presented in the Results section are likely explained by differences in the methods used. While our study used filter paper and incubated the dishes at 60% RH, the previous study used sand to cover the bottom of Petri dishes with 7% RH, which might have hindered EPN activity as they need moisture to survive and move around [25]. The authors also only tested one nematode concentration of 2000 nematodes/mL, while here various concentrations of up to 40,000 nematodes/mL were tested.

Previous work has also tested commercial formulations of *S. feltiae*, *S. carpocapsae*, *H. bacteriophora* and *H. megidis* (Poinar, Jackson & Klein) against *Phyllotreta* spp. flea beetle adults [54]. Here, the authors tested concentrations of 2000, 10,000 or 20,000 nematodes/mL and at three temperatures in Petri dishes on filter paper and found that for all EPN treatments, flea beetle mortality was higher than in the water control treatment. They observed that *S. feltiae* caused the highest mortality at every assessment date, while *H. bacteriophora* was the most effective when only the end of the experimental period was considered, after eight days. It was also reported that EPNs were in general more effective with increasing temperature. Indeed, temperature had a greater effect on flea beetle mortality than changing EPN concentration. Results presented here against CSFB are similar to those reported previously for *Phyllotreta* spp.; however, the role of temperature should be included in future studies with CSFB, as well as testing whether incubating the Petri dishes in the dark would produce different results. The fact that these EPN species seem to be more effective at higher temperatures is an important finding, as winter oilseed rape crops grown in the UK and northern European countries are at their most vulnerable growth stage in autumn when temperatures are declining.

Two recent studies have tested the efficacy of EPNs against the striped flea beetle [38,64]. Both studies produced results broadly similar to the results reported in the present study. For example, all species of EPN tested were found to successfully infect and kill the striped flea beetle [38]. Four days after treatment application, *S. carpocapsae* at a concentration of 50 nematodes/insect caused more than 50% adult mortality. Five days after treatment, 79–83% adult mortality was recorded at 200 nematodes/insect with *S. carpocapsae* and *S. siamkayai*. These results appear to be broadly similar to the results reported in the present study.

Testing the efficacy of EPN under field conditions is important to ensure that results from the laboratory can be translated successfully. While several field studies have reported encouraging results using EPNs against various flea beetle species such as CSFB [23,63], unspecified *Phyllotreta* species [33,41,42] and crucifer flea beetle and striped flea beetle [34,36,38,39,40], these studies have not been based on initial laboratory testing results.

Future studies should test a range of temperatures relevant to crop conditions when evaluating EPN efficacy against CSFB. A recent study stated that larval stages of CSFB may be a better target for EPN [63], but more work is required using potted plants infested with larvae under laboratory conditions to provide further information on the suitability of this growth stage of the target pest. There is also evidence that a specific strain of *H. bacteriophora*, SDT1-IL1, was more effective than the commercial *H. bacteriophora* Nematop^®^ at killing CSFB larvae (60% mortality versus 30%); so, using different strains of EPN could also be a potential focus of future studies.

### 4.2. Compatibility between Entomopathogenic Nematodes and Adjuvants

Overall, the adjuvants tested with *S. feltiae* and *H. bacteriophora* were compatible over a period of seven days of exposure, except for glycerin which affected *S. feltiae* negatively at the highest concentration tested, and the flame retardant, which negatively affected *H. bacteriophora* survival as concentrations increased. In the case of these two adjuvants, it appears that nematode survival is linked to concentration. A study [47] working with *H. indica* Poinar, Karunakar and David, a nematode species closely related to *H. bacteriophora*, reported 81% survival of nematodes after two hours of exposure to 0.1% glycerin, which was significantly higher than nematode survival when exposed to other adjuvants tested (Triton X-100, paraffin liquid, castor oil, palm oil and sunflower oil). However, no nematodes survived more than eight hours of contact with any of the adjuvants, while in the present study 100% of *H. bacteriophora* survived for at least seven days when exposed to the same concentration of glycerin.

As evidence of the potential importance of developing suitable EPN–adjuvant combinations, it is useful to consider other pests. For example, to control the lesser peachtree borer, *Synanthedon pictipes* Grote and Robinson (Lepidoptera: Sesiidae), two studies [49,50] applied *S. carpocapsae* alongside Barricade^®^ (Barricade International (firegel.com) (accessed on 11 April 2023)), a sprayable polymer gel typically used as a fire retardant and similar in composition to the fire retardant used in the present study, and compared it with chlorpyrifos. The authors concluded that applying *S. carpocapsae* and 2% Barricade^®^ at the same time was more effective than applying them separately or not treating, and that the combination was at least as effective as chlorpyrifos. These results contrast with the results reported here where the flame retardant was not compatible with *H. bacteriophora*.

Based on these previous studies, researchers have tested commercial formulations of *S. feltiae* and *S. carpocapsae* [45] against crucifer flea beetle adults in canola fields combined with 1% Barricade^®^, or with an imidacloprid insecticidal product (Gaucho). *Steinernema feltiae* was only found to be effective in reducing leaf damage by adult beetles and improving yield when combined with the Barricade^®^ or when combined with imidacloprid. One other advantage of polymer gels like Barricade^®^ or the fire retardant product used in the present study is that they are non-toxic; hence, they do not have negative environmental impacts.

The next steps for future studies would be to explore whether the adjuvants tested in the present study are effective in improving nematode persistence and application on oilseed rape plants under field conditions. These results give encouragement that the apparent compatibility of Flametect Nitro D with *S. feltiae*, as shown in this study, should be progressed to testing under more realistic environmental conditions.

## Figures and Tables

**Table 1:** Entomopathogenic nematode (EPN) products used in the cabbage stem flea beetle (CSFB) mortality bioassay. Temperature range refers to the soil temperature indicated on the product label. All packs contained 5 × 10^7^ individuals. The percentage values represent the proportion of nematodes in relation to the inert carrier contained in each pack.

Product Name	Manufacturer	Nematode Species	Temperature Range
Nemasys^®^	BASF Agricultural Solutions, Littlehampton, UK	*Steinernema feltiae* (90%)	10–30 °C
Nemasys^®^ C		*Steinernema carpocapsae* (87%)	12–30 °C
Nemasys^®^ L		*Steinernema kraussei* (88%)	5–30 °C
Nemasys^®^ H		*Heterorhabditis bacteriophora* (82%)	12–30 °C

**Table 2:** Adjuvants tested alongside entomopathogenic nematodes (EPNs).

Product Name	Manufacturer	Active Ingredients	Concentrations	Replicates
Flametect Nitro D	Eco-Sol Ltd., Barry, UK	Nitrogen-based solution (34% minimum), polymer-binder system (30%)	0.01, 0.1, 1 and 10%	3 per concentrations
Xanthan gum	Sigma-Aldrich, St Louis, MO, USA	Xanthan gum from *Xanthomonas campestris*	0.001, 0.01, 0.1 and 1%	3 per concentration
Glycerin	Fisher Scientific, Loughborough, UK	Glycerol ≥ 99%	0.01, 0.1, 1 and 10%	3 per concentration

## Data Availability

The datasets used and/or analysed during the current study are available from the corresponding author on request.
